# Culture-independent analyses of carrion beetle (Coleoptera: Silphidae) secretion bacterial communities

**DOI:** 10.1128/spectrum.01694-23

**Published:** 2023-10-24

**Authors:** Carrie J. Pratt, Casey H. Meili, Noha H. Youssef, W. Wyatt Hoback

**Affiliations:** 1 Department of Microbiology and Molecular Genetics, Oklahoma State University, Stillwater, Oklahoma, USA; 2 Department of Entomology and Plant Pathology, Oklahoma State University, Stillwater, Oklahoma, USA; University of Minnesota Twin Cities, St. Paul, Minnesota, USA

**Keywords:** Silphidae, *Nicrophorus*, burying beetle, carrion beetle

## Abstract

In the central plains of North America, the beetle family Silphidae comprised two subfamilies, Silphinae and Nicrophorinae, differentiated by reproductive behaviors. Silphinae, known as carrion beetles, feed on carrion and fly larvae and produce free-living larvae that receive no parental care. Adult Nicrophorinae, known as burying beetles, prepare a vertebrate carcass into a brood ball and provide biparental care to their offspring. Preparation of a brood ball involves coating the carcass in antimicrobial oral and anal secretions. These secretions contain a community of microbes, referred to as the secretion microbiome, which inhibit carcass microbe succession, preventing normal decomposition. Here, the secretion microbiomes of five species of Nicrophorinae and two species of Silphinae, both sampled from Oklahoma, with additional Nicrophorinae from Nebraska, were characterized using culture-independent analyses to understand and decipher factors shaping diversity and community structure. We identify the core secretion microbiome across Silphidae and show that, while the host subfamily, secretion type, and collection locality had no significant effect on the bacterial community alpha diversity, these factors significantly influenced bacterial community structure. Global and local tests of phylogenetic associations identified 14 genera with phylogenetic signals to the host subfamily and species. Description of the bacterial communities present in silphid secretions furthers our understanding of how these beetles interact with microbes for carcass nutrient processing. Future culture-dependent studies from silphid secretions may identify novel antimicrobials and nontoxic compounds that can act as meat preservatives or sources for antimicrobials.

**IMPORTANCE:**

The manuscript explores the secretion bacterial community of carrion and burying beetles of the central plains of North America. A core secretion microbiome of 11 genera is identified. The host subfamily, secretion type, and collection locality significantly affects the secretion microbiome. Future culture-dependent studies from silphid secretions may identify novel antimicrobials and nontoxic compounds that can act as meat preservatives or sources for antimicrobials.

## INTRODUCTION

Beetles in the central plains of North America belonging to the Silphidae subfamilies Silphinae and Nicrophorinae are differentiated by morphology and reproductive behaviors. The subfamily Silphinae contains carrion beetles that oviposit on or near a carcass and hatch free-living larvae that do not receive parental care. Adults and larvae feed on both the carcass and maggots that are also feeding on the carcass. The subfamily Nicrophorinae contains burying beetles, which exhibit a unique reproductive strategy involving the preparation of a small carcass into a brood ball and provision of biparental care to offspring. While the two subfamilies of Silphidae are estimated to have split at ~113 Mya (105.6–172.9) (1, 2), implying that complex parental care in beetles evolved during the Mesozoic, some fossil records (3) during the Cretaceous indicate that a simple form of parental care might have evolved earlier as what is known from the extant *Ptomascopus* species (3). Adult members of Nicrophorinae coat a brood ball with oral and anal secretions that exhibit antimicrobial characteristics and prevent the microbial succession of soil and carcass microbes that would normally cause the carcass to decompose (4 – 8). This community of secretion microbes makes up the secretion microbiome, which is distinct from the gut microbiome, the microbiome of the carcass, and the microbiome of a prepared carcass (9).

The antimicrobial activities of secretions differ among members of the Silphidae subfamilies Silphinae and Nicrophorinae, where, in general, nicrophorines produce secretions with antimicrobial activity while silphines do not (10). While it was previously shown that antimicrobial peptides and lysozymes are produced by the beetles themselves (*Nicrophorus vespilloides*) (11), we hypothesize that substantially different secretion microbiomes between the beetle subfamilies may indicate that some antimicrobial activity derives from endosymbiotic bacteria. Previous research on European and North American silphid gut microbiomes showed that bacterial communities are more congruent with sampling locality than host phylogeny (6). Thus, sampling beetles from the same locality may reveal bacterial communities that more strongly reflect silphid host phylogeny. Finally, Hoback et al. (10) found that antimicrobial activities differed between oral and anal secretions. Identifying differences in the microbiomes released in oral and anal secretions may help identify reasons for antimicrobial activity differences.

Five Nicrophorinae species all belonging to the genus *Nicrophorus* (*Nicrophorus americanus, N. tomentosus*, *N. orbicollis*, *N. marginatus,* and *N. pustulatus*) were included in this research. One of these five species, the American burying beetle (*Nicrophorus americanus*; ABB), was placed on the US state and federal endangered species lists in 1989 (https://ecos.fws.gov/ecp/report/species-listings-by-year?year=1989). Four of these five species*, N. americanus, N. orbicollis, N. marginatus,* and *N. tomentosus,* prepare a brood ball for their offspring and exhibit biparental care throughout larval development (4 – 7). Preparation of a brood ball includes coating it in antimicrobial secretions that contain bacteria that modify the microbial communities of the carcass, making it usable by the beetles and their offspring (12). In contrast, *N. pustulatus* has undergone a host shift from carrion to snake eggs (13) and lacks secretions with antimicrobial activity (10).

Two Silphinae species, *Necrodes surinamensis* and *Necrophila americana,* were also included. These species provide no parental care and do not prepare a brood ball (8). Adults feed primarily on maggots at carcasses, but will also feed on carrion (8). Females oviposit on or near the carcass and free-living larvae hatch after 2–4 days (8). *Necrodes surinamensis* is unusual in that it possesses antimicrobial defensive anal secretions hypothesized to be the first evolutionary steps toward the antimicrobial secretions for brood ball preparation found in *Nicrophorus* spp. (10).

Characterizing the previously undescribed bacterial component of secretion microbiomes of several species within Silphidae allows for the identification of similarities and differences among Silphinae and Nicrophorinae and by secretion source. We hypothesized that the bacterial microbiomes of the Silphinae and the Nicrophorinae secretions would have differences in community structure that would correspond with either the phylogenies of the subfamily or reflect differences in geographic location or secretion source.

## MATERIALS AND METHODS

### Beetle and secretion collection

Oral and anal secretions were separately collected from seven silphid species. One male and one female *Nicrophorus americanus* (*n* = 2) and mixed sex *N. tomentosus* (*n* = 4), *N. orbicollis* (*n* = 4), *N. pustulatus* (*n* = 4), *Necrodes surinamensis* (*n* = 5), and *Necrophila americana* (*n* = 5) were collected from the same above-ground pitfall trap (14) baited with rotten rat in June 2020 at Camp Gruber, Oklahoma (35.737739–95.146594). Secretions were collected on site before beetles were released. One male and one female *Nicrophorus americanus* (*n* = 2) and mixed sex *N. marginatus* (*n* = 5) were trapped in August 2020 near O’Neill, Nebraska and brought back to Oklahoma State University where their secretions were collected. All secretions were collected on sterile cotton swabs that were then broken off at the tip into sterile 1.5 mL microcentrifuge tubes and frozen until DNA extraction. At the time of sampling, *Nicrophorus americanus* (the ABB) was on the US state and federal endangered species lists (https://ecos.fws.gov/ecp/report/species-listings-by-year?year=1989) [although it was down-listed from endangered to threatened a few months following sampling (15)]. In the current study, ABB was the only species of beetle collected from both Oklahoma and Nebraska. Also, ABB is the only species of beetle (out of all seven studied here) where individual male and female sexes could readily be differentiated. Finally, ABBs are the largest silphids in North America, and accordingly, their secretions tend to be more copious than other species secretions. For all the above reasons, secretions from *Nicrophorus americanus* beetles from each state and from each sex were treated individually rather than being pooled together, while secretions from all individuals belonging to the other beetle species were pooled (with anal and oral secretions kept separate). Overall, a total of 31 individuals were sampled. For each individual beetle, an oral and an anal swab was collected. Oral secretion and anal secretions from *N. tomentosus*, *N. orbicollis*, *N. pustulatus*, *Necrodes surinamensis*, *Necrophila americana,* and *N. marginatus* were individually pooled (for a total of six oral and six anal secretions representing these six species), while the secretions from the four individuals belonging to *Nicrophorus americanus* (a male and a female from OK, and a male and a female from NE) were not pooled (for a total of four oral and four anal secretions representing this single species). These 20 samples (Table S1; 10 oral and 10 anal secretions; 6 NE and 14 OK secretions) were then used for DNA extraction, PCR amplification, and sequencing as explained below. While all samples were collected during the breeding season, however, since finding buried carcasses is usually difficult in field settings, we acknowledge that the collections might not have occurred during breeding, where the secretions are typically more abundant and the lytic activity is upregulated as shown before (11, 12).

### DNA extraction, PCR amplification, and Illumina sequencing

DNA was extracted from secretions using a DNeasy Plant Pro kit (Qiagen) according to manufacturer instructions. Resulting DNA concentrations were quantified using a Qubit fluorometer (Life Technologies, Carlsbad, CA). Isolated DNA was then used as a template to PCR amplify the 16S rRNA V4 hypervariable region using the 515F and 806R prokaryotic-specific primer pair (16) modified to include the Illumina overhang adaptors. PCR reactions contained 2 µL of DNA, 25 µL of the DreamTaq 2X master mix (Life Technologies, Carlsbad, California), and 2 µL of each primer (10 µM) in a 50 µL reaction mix. The PCR protocol consisted of an initial denaturation for 5 min at 95°C followed by 40 cycles of denaturation at 95°C for 1 min, annealing at 55°C for 1 min and elongation at 72°C for 30 s, and a final extension of 72°C for 10 min. A negative control reaction (reagent-only control) was run at the same time and resulted in no amplification. PCR products were cleaned using PureLink PCR cleanup kit (Life Technologies, Carlsbad, California), and the clean product was used in a second PCR reaction to attach the dual indices and Illumina sequencing adapters using Nextera XT index kit v2 (Illumina Inc., San Diego, California). These second PCR products were then cleaned using PureLink gel extraction kit (Life Technologies, Carlsbad, California), individually quantified, and pooled using the Illumina library pooling calculator (https://support.illumina.com/help/pooling-calculator/pooling-calculator.htm) to prepare a 100 pM library that was then sequenced using the paired-end Illumina iSeq-100 sequencing system.

### Sequence processing

The software package mothur v.44 was used for sequence processing and analysis, with most steps derived from the MiSeq SOP available from the mothur website (17). Forward and reverse sequence pairs were assembled into contigs that were further processed to eliminate sequences with ambiguous bases, sequences longer than 300 bp or shorter than 260 bp, and sequences with homopolymer stretches longer than 8 bp. This resulted in a total of 928,947 high-quality sequences from all samples. Sequences were aligned in mothur using the recreated Silva seed alignment database as a template, and alignments were pre-clustered and de-noised using a pseudo-single linkage algorithm (18). Misaligned and possible chimeric sequences were removed using chimera.slayer in mothur. The remaining sequences were classified in mothur using the Silva (V. 132) taxonomic outline, and the resulting taxonomy file was used to cluster sequences into operational taxonomic units (OTUs) at the genus level using the command phylotype in mothur. The list file obtained was then used to create a shared file (using make.shared in mothur) that was subsequently used for all downstream analyses. Because amplicons analyzed were 300 bp long, we opted to classify sequences based on their taxonomy down to the genus level only, rather than species or strain level, for confidence of assignment.

### Factors impacting the secretions alpha diversity and community structure

We considered two types of factors that could potentially impact secretion diversity and community structure: host-associated factors and non-host-associated factors. For host-associated factors, we opted for testing the effect of host subfamily (Nicrophorinae versus Silphinae), rather than host species, to account for the shortcoming of having only one pooled sample representing each species (except for the ABB). For non-host-associated factors, we considered secretion source (anal versus oral) with the addition of an interaction term (subfamily-specific differences in the secretion microbiome) as explained below, and the state of origin (OK versus NE) with the addition of a nestedness term as explained before.

### Alpha diversity measures

Alpha diversity estimates (observed number of genera, Chao, Abundance-based Coverage Estimator (ACE), Shannon, Simpson, inverse Simpson, and Fisher alpha diversity indices) were calculated using the command estimate_richness in the phyloseq (v1.42.0) R package. The importance of various factors (host subfamily, state of origin, and secretion source) in shaping the observed patterns of alpha diversity was examined using analysis of variance (calculated using the aov command in R v4.2.2). Rarefaction curve analysis was performed in mothur.

### Community structure

The genus-level shared file created in mothur was used to calculate both dissimilarity matrix-based (e.g., Bray-Curtis) as well as phylogenetic similarity-based (weighted UniFrac) beta diversity indices using the ordinate command in the phyloseq (v1.42.0) R package. The pairwise values were used to construct ordination plots (both Principal Cordinate Analysis (PCoA) and Non-metric Multidimensional Scaling (NMDS)) using the function plot_ordination in the phyloseq R package. Bacterial taxa were also plotted on the same ordination plots. To partition the dissimilarity among the sources of variation (host subfamily, state of origin, and secretion source), PERmutational Multivariate ANalysis Of VAriance (PERMANOVA) tests (19) were run for each of the above beta diversity measures using the vegan (v 2.6-4) command Adonis, with the addition of nestedness (host subfamily nested in state), and interaction (to test for subfamily specific differences in the secretion microbiome) terms. The F-statistics *P*-values obtained were compared to identify the factors that significantly affect the secretion community structure, and the percentage variance explained by each factor was calculated as the percentage of the sum of squares of each factor to the total sum of squares.

To further quantitatively assess these factors in explaining community structure, we used three multivariate regression approaches based on matrices comparison: multiple regression of matrices (MRM), Mantel tests for matrices correlations, and Procrustes rotation. For these multivariate analyses, two microbial community dissimilarity matrices were calculated based on Bray-Curtis (calculated from the genus shared file using vegdist command in vegan), and UniFrac weighted (calculated using the distance command in the phyloseq package), and compared to a matrix of each of the factors tested (host phylogeny, state of origin, and secretion source). For the host phylogeny, a cophenetic matrix was calculated [using the command cophenetic in the ape (V 5.6-2) R package] based on the Newick tree downloaded from timetree.org (20) and modified to include all the samples studied here with very short branch length between samples from the same beetle species (shown in Fig. S1). For the state of origin and the secretion source, since these were nominal values, matrices were constructed by Gower transformation (21). The two community dissimilarity matrices (Bray-Curtis-based and UniFrac weighted-based) were each then correlated to each of the factor matrices (*n* = 3) using the commands MRM, and mantel in the ecodist (V 2.0.9) R package, for running multiple regression on matrices, and Mantel tests, respectively. The Procrustes rotation was calculated using the protest command in the vegan R package. For each of the factors tested, six total correlations (three multivariate regression methods × two dissimilarity indices ) were compared to evaluate the importance of the factor tested in explaining the secretion bacterial community structure. First, the *P*-values were evaluated for significance of correlation, followed by comparing coefficients (*R* ^2^ regression coefficients of the MRM analysis, Spearman correlation coefficients of the Mantel test, and symmetric orthogonal Procrustes statistic of the Procrustes analysis) for the importance of the factor tested in explaining community structure.

### Identifying bacterial taxa contributing to community structure differences

To identify bacterial genera differentially abundant in one host subfamily, state of origin, or secretion type, we used the genus-level shared file in mothur to calculate both linear discriminant analysis (LDA) effect size (LEfSe) and Metastats. Genera with calculated LDA scores and/or significant Metastats *P*-values were considered differentially abundant. For pinpointing specific beetle species/subfamily-bacterial associations, we calculated three global phylogenetic signal statistics, Abouheif’s C_mean_, Moran’s I, and Pagel’s lambda using the phyloSignal command in the phylosignal (V 1.3) R package. We considered any genus with *P*-value <0.05 with at least one statistic to be significantly correlated to the host phylogenetic tree. Next, to calculate LIPA (local indicator of phylogenetic association) values for each sample-genus pair, we used the lipaMoran command in the phylosignal R package. Genera with LIPA *P*-values <0.05 were considered significantly phylogenetically associated with a host species/subfamily. We considered average LIPA values in the range of 0.2–0.4 to represent weak associations, in the range of 0.4–1 to represent moderate associations, and above 1 to represent strong associations.

## RESULTS

### Overall bacterial community composition

We analyzed 20 samples from 21 *Nicrophorinae* and 10 *Silphinae* individuals (Fig. S1). These included 10 anal and 10 oral samples. Six of the samples were collected from Nebraska, while 14 were collected from Oklahoma. Analysis was conducted to give a detailed view of the community inhabiting the oral and anal secretions of these beetles and to understand and decipher factors shaping the microbiome diversity and community structure.

A total of 928,947 sequences were obtained after quality control (average 41,813 sequences/sample). Good’s coverage values of 99.8%–99.99% suggest the majority of the community was sampled.

**Fig 1:**
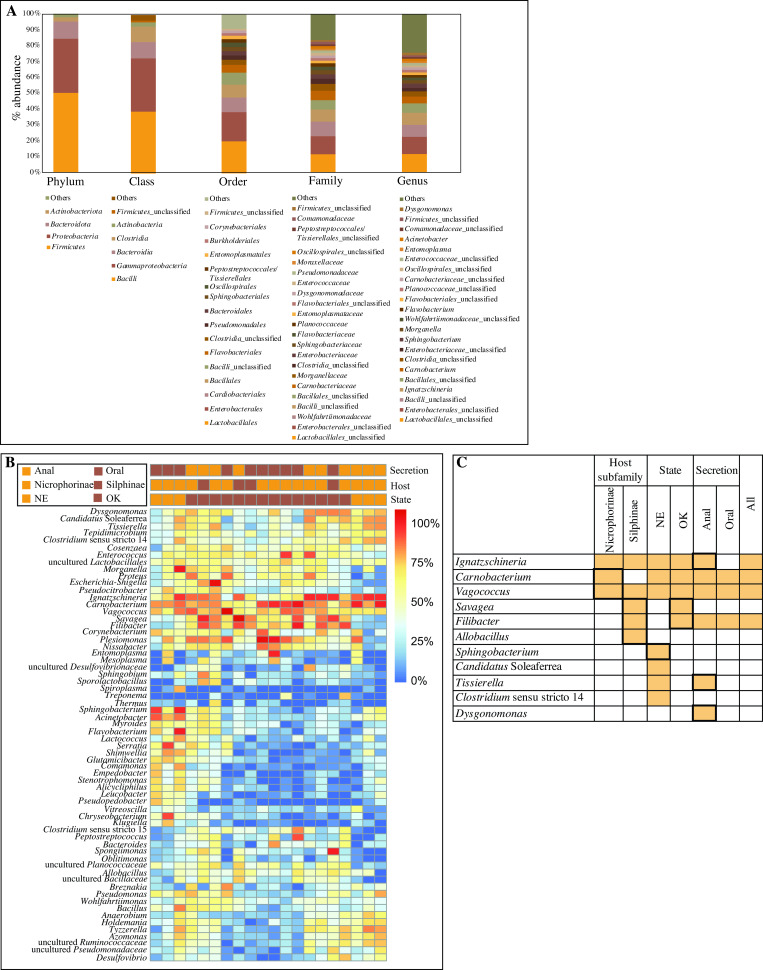
Total bacterial community composition in the samples studied. (**A**) Stacked bar chart with the total distribution of bacteria phyla, classes, orders, families, and genera. Taxa with total number of sequences >10,000 are displayed, with the remainder lumped as “Others.” The legend at the bottom is shown in the same order of the stacked bars from bottom to top. (**B**) Heatmap of the distribution of the 64 most abundant genera (also shown in A) across samples. Each column represents one sample. The host subfamily, state of origin, and secretion type for each sample are color coded and displayed above the heatmap. (**C**) Genera of the core microbiome. Orange cells represent genera found in 70% of samples with >1% abundance. Bold outlines indicate that the genus abundance was significantly higher compared to the paired counterpart.

Overall, 36 phyla, 92 classes, 241 orders, 440 families, and 1,452 genera were identified (Fig. 1A). The community at the phylum level was dominated by Firmicutes, Proteobacteria, and Bacteroidetes, which collectively represented 95.58% of the total community. The majority of Firmicutes sequences belonged to the families *Planococcaceae*, *Carnobacteriaceae*, *Vagococcaceae*, *Ruminococcaceae*, *Enterococcaceae*, and unclassified families in the orders *Peptostreptococcales-Tissierellales*. Proteobacteria sequences were largely identified as belonging to the Gamma-Proteobacteria families *Wohlfahrtiimonadaceae*, *Enterobacteriaceae*, *Morganellaceae*, and *Pseudomonadaceae*. The families *Flavobacteriaceae*, *Sphingobacteriaceae*, and *Dysgonomonadaceae* constituted the majority of the sequences belonging to the phylum Bacteroidetes (Fig. 1A). Within these families constituting the majority of the bacterial community in the samples studied, 64 genera with >1% total abundance were identified (Fig. 1B). Eleven genera were identified as the “core microbiome” (defined as the genera present in at least 70% of all samples with at least 1% abundance) (Fig. 1C). These genera are *Ignatzschineria, Carnobacterium, Vagococcus, Savagea, Filibacter, Allobacillus, Sphingobacterium, Candidatus Soleaferrea, Tissierella, Clostridium sensu stricto 14,* and *Dysgonomonas*.

### Factors impacting bacterial diversity

**Fig 2:**
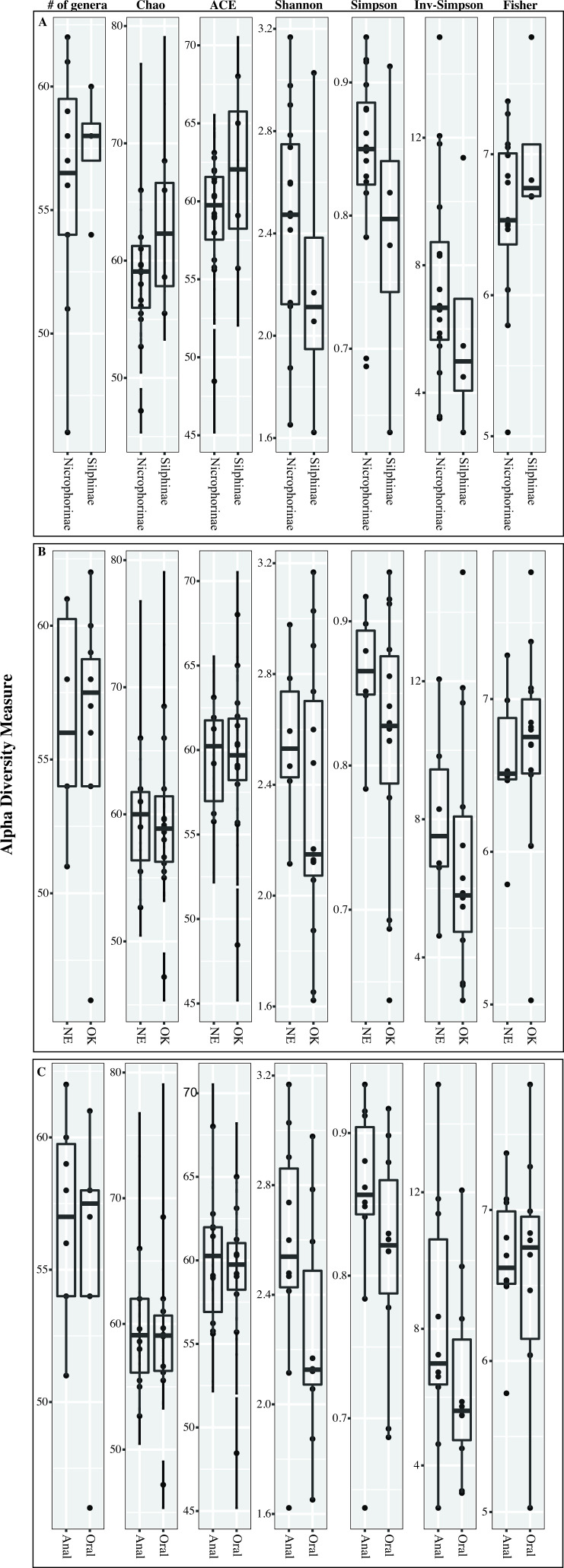
Alpha diversity measures across the samples studied shown as box and whisker plots (displayed on top: total number of genera observed, Chao diversity index, ACE richness index, Shannon diversity index, Simpson’s diversity index, inverse Simpson’s diversity index, and Fisher’s alpha diversity index) classified by (**A**) host subfamily, (**B**) state of origin, and (**C**) secretion type. The indices were calculated using the function ordinate in the phyloseq package, and the plots were generated using plot_ordination function in phyloseq.

Multiple measures were used to compare alpha diversity across samples (Fig. 2; Fig. S2). Across all samples, no significant differences were observed in alpha diversity measures when comparing samples from different secretion types (oral versus anal), different host subfamilies (*Nicrophorinae* versus *Silphinae*), or samples originating from different states (OK versus NE) (Student’s *t*-test *P*-value >0.1). Rarefaction analysis showed similar patterns of alpha diversity (Fig. S2).

### Factors impacting community structure

**Fig 3:**
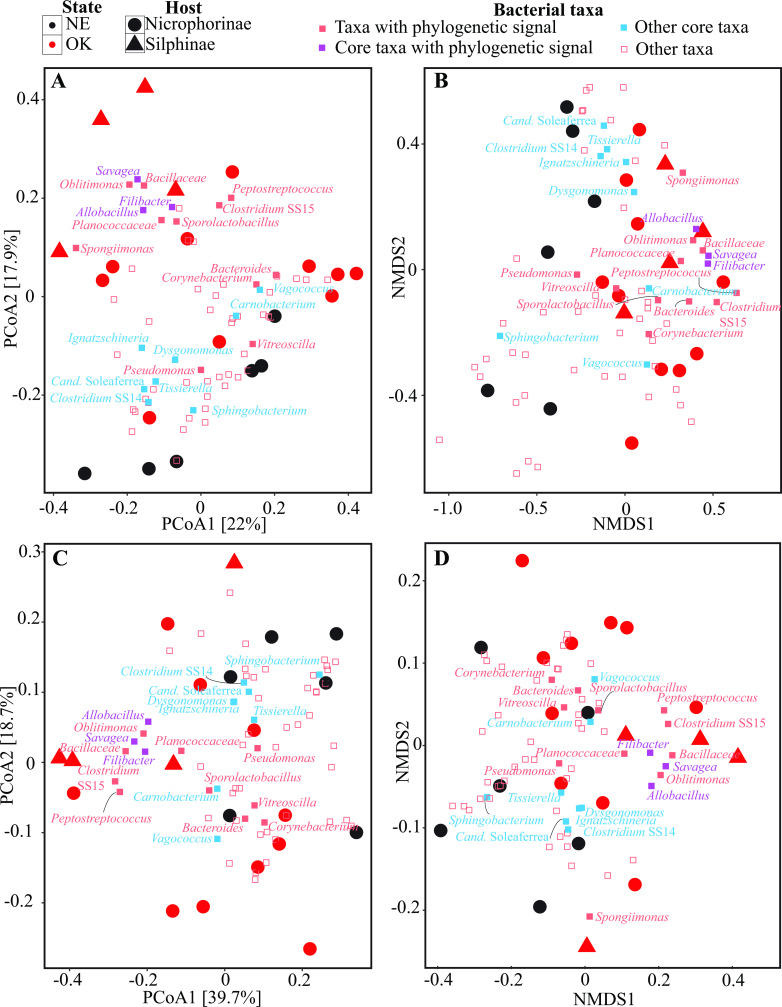
Bacterial community structure in the samples studied. Ordination plots (both PCoA and NMDS) constructed with both dissimilarity matrix-based [Bray-Curtis (A and B)] and phylogenetic similarity-based [weighted UniFrac (C and D)] indices describe the similarity between communities. The percentage variance explained by the first two PCoA axes are shown on the corresponding axis. Samples are color coded by the state of origin, while the shape depicts the host subfamily as shown on top. Biplots with the 64 most abundant bacterial taxa are shown in Fig. S3.

PCoA and NMDS plots constructed using both dissimilarity matrix-based (Bray-Curtis) as well as phylogenetic similarity-based (weighted UniFrac) indices explained 39.9%–58.4% of variance between samples (Fig. 3; Fig. S2). PERMANOVA analysis conducted to partition the dissimilarity among the sources of variation (sample state of origin, secretion type, and host subfamily) showed that, regardless of the beta diversity measure used, both all factors significantly explained diversity (F-statistics *P*-value <0.04), with the state explaining the most variance (12.6%–16.3% depending on the index used), followed by the host subfamily (explaining 10.1%–11.1% of variance depending on the index used), and the secretion type (explaining 10%–10.8% of variance depending on the index used). The nestedness of subfamily in state was found to be significant [F-statistics *P*-value = 0.008 (Bray-Curtis) to 0.011 (weighted UniFrac)], and the interaction of subfamily with the secretion type was only found to be significant with Bray-Curtis (F-statistics *P*-value = 0.037), where the interaction of these two factors explained 7.7% of variance, but not with weighted UniFrac (F-statistics *P*-value = 0.277).

To further quantitatively assess factors that explain beta diversity, we used three multivariate analysis methods (multiple regression of matrices, Mantel tests for matrices correlations, and Procrustes rotation) to compare the dissimilarity matrices (Bray-Curtis and weighted UniFrac) to a matrix of each of the factors tested (sample state of origin, secretion type, and host genus). Results of matrices correlation (six total correlations; three methods × two dissimilarity indices) using each of the three methods, and regardless of the index used, confirmed the importance of host subfamily and state of origin in explaining the beetles’ secretion community structure (Table 1). Host state of origin was found to be significant in all six correlations (*P*-value <0.03), while the host subfamily was found to be significant in five out of the six correlations (*P*-value <0.05). Secretion type was only found to be significant (*P*-value <0.01), when Bray-Curtis was used as the beta diversity measure (i.e., in three out of the six correlations). Furthermore, comparing the correlation coefficients produced by each of the methods showed that the state of origin and the host subfamily equally explain the community structure (as evident by similar *R* ^2^ regression coefficients of the MRM analysis, the similar Spearman correlation coefficients of the Mantel test, and the similar symmetric orthogonal Procrustes statistic of the Procrustes analysis). In contrast, secretion type, in the three significant correlations, showed lower correlation coefficients indicating a lower overall effect on community structure compared to the state of origin and the host subfamily.

**TABLE 1:** Results of community structure matrices correlation [six total correlations for each factor studied (row); three multivariate analysis methods x two dissimilarity indices]^*a*^

Diversity index	Bray-Curtis						Weighted UniFrac					
Multivariate analysis test	MRM		Mantel		Procrustes		MRM		Mantel		Procrustes	
	Regression coefficient	*P*-value	Mantel coefficient	Two-tailed *P*-value	Coefficient	Significance	Regression coefficient	*P*-value	Mantel coefficient	Two-tailed *P*-value	Coefficient	Significance
State	0.074	**0.009**	0.238	**0.016**	0.343	**0.011**	0.069	**0.015**	0.217	**0.027**	0.370	**0.028**
Subfamily	0.070	**0.038**	0.208	0.092	0.360	**0.008**	0.089	**0.013**	0.279	**0.021**	0.418	**0.010**
Secretion	0.053	**0.008**	0.174	**0.009**	0.401	**0.002**	0.031	0.057	0.089	0.097	0.308	0.106
MRM full model	0.149	**0.005**					0.150	**0.006**				

^*a*^ For each multivariate analysis method x dissimilarity index used, values are shown for the method’s correlation coefficient (symbolizing the strength of correlation) and *P*-value (symbolizing the significance of correlation). Significant correlations are shown in bold text (*P* < 0.05). Results of the MRM full model are also shown for each of the diversity indices used.

### Bacterial taxa contributing to community structure differences

Community structure analysis above suggested that host subfamily and state of origin are important in shaping the bacterial community. In order to identify differentially abundant genera contributing to the differences observed in community structure across samples, we used LEfSe and Metastats (Table 2). Analyses showed that several genera from the core microbiome were differentially abundant when comparing host subfamilies and state of origin, as well as when comparing anal to oral secretions. The genera *Carnobacterium, Vagococcus, Clostridium sensu stricto 14*, and *Tissierella* were significantly more abundant within the secretion bacterial communities of members of the subfamily Nicrophorinae, while the genera *Filibacter*, *Savagea,* and *Allobacillus* were significantly more abundant within the subfamily Silphinae. When comparing state of origin, the core genera *Filibacter* and *Savagea* were found to be significantly more abundant (among other genera) within the secretion bacterial communities of beetles from Oklahoma, while the core genus *Sphingobacterium* was more abundant in beetles secretions from Nebraska. Finally, the core genera *Ignatzschineria, Candidatus Soleaferrea, Tissierella, Clostridium sensu stricto 14,* and *Dysgonomonas* were found to be more significantly abundant in the anal secretions. These patterns were also clear in the ordination biplots (Fig. S3).

**TABLE 2:** Results of linear discriminant analysis (LDA) effect size (LEfSe) and Metastats analysis to identify abundant taxa that contribute to the differences observed among community structure across samples^*a*^

Subfamily (Silphinae vs Nicrophorinae)						
Genus	Silphinae	Nicrophorinae	Class	LEfSe		Metastats
				LDA	*P*-value	*P*-value
*Carnobacterium*	1.87 ± 0.83	**12.04 ± 2.60**	Nicrophorinae	4.816	**0.006**	**0.001**
*Allobacillus*	**1.74 ± 0.45**	0.24 ± 0.09	Silphinae	3.853	**0.005**	**0.004**
*Savagea*	**30.19 ± 8.59**	2.29 ± 0.87	Silphinae	5.127	**0.002**	**0.004**
*Tissierella*	0.16 ± 0.08	**1.68 ± 0.47**	Nicrophorinae	–	–	**0.005**
*Tyzzerella*	0.12 ± 0.07	**1.52 ± 0.58**	Nicrophorinae	–	–	**0.028**
*Clostridium sensu stricto 14*	0.14 ± 0.02	**1.11 ± 0.41**	Nicrophorinae	–	–	**0.029**
Uncultured *Bacillaceae*	**1.18 ± 0.57**	0.10 ± 0.05	Silphinae	3.825	**0.005**	0.079
*Filibacter*	**12.8 ± 4.29**	4.23 ± 1.75	Silphinae	4.370	**0.030**	0.086
*Vagococcus*	1.75 ± 0.35	**7.37 ± 3.05**	Nicrophorinae	4.403	**0.033**	0.089
Uncultured *Planococcaceae*	**1.06 ± 0.46**	0.36 ± 0.12	Silphinae	3.795	**0.030**	0.198
*Spongiimonas*	**7.27 ± 6.47**	0.09 ± 0.05	Silphinae	4.675	**0.008**	0.391

State (Oklahoma vs Nebraska)						
Genus	OK	NE	Class	LEfSe		Metastats
				LDA	*P*-value	*P*-value
*Filibacter*	**8.48 ± 2.21**	0.02 ± 0.01	OK	4.590	**0.001**	**0.001**
*Savagea*	**11.23 ± 4.08**	0.04 ± 0.01	OK	4.793	**0.001**	**0.005**
*Proteus*	**2.15 ± 0.84**	0.16 ± 0.10	OK	4.081	**0.013**	**0.015**
*Enterococcus*	**3.77 ± 1.78**	0.36 ± 0.22	OK	4.548	**0.021**	**0.048**
*Glutamicibacter*	0.04 ± 0.02	**1.07 ± 0.60**	NE	3.542	**0.006**	0.074
*Acinetobacter*	0.19 ± 0.11	**3.66 ± 2.03**	NE	4.335	**0.003**	0.076
*Sphingobacterium*	0.21 ± 0.11	**5.61 ± 3.37**	NE	4.606	**0.048**	0.099
*Peptostreptococcus*	**1.05 ± 0.71**	0.01 ± 0.01	OK	3.770	**0.017**	0.151
*Shimwellia*	0.08 ± 0.05	**1.30 ± 0.85**	NE	3.324	**0.020**	0.158
*Escherichia-Shigella*	**2.84 ± 2.10**	0.06 ± 0.03	OK	3.780	**0.004**	0.202
*Spongiimonas*	**2.18 ± 1.89**	0.01 ± 0.00	OK	4.215	**0.010**	0.300

Secretion type (oral vs anal)						
Genus	Oral	Anal	Class	LEfSe		Metastats
				LDA	*P*-value	*P*-value
*Ignatzschineria*	4.16 ± 2.01	**14.74 ± 2.91**	Anal	4.738	**0.005**	**0.007**
*Tissierella*	0.22 ± 0.12	**2.54 ± 0.61**	Anal	4.089	**0.001**	**0.001**
*Tyzzerella*	0.23 ± 0.18	**2.25 ± 0.85**	Anal	3.896	**0.002**	**0.007**
*Candidatus Soleaferrea*	0.46 ± 0.25	**2.86 ± 0.80**	Anal	4.048	**0.007**	**0.008**
Uncultured *Ruminococcaceae*	0.12 ± 0.07	**1.03 ± 0.42**	Anal	3.736	**0.007**	**0.031**
*Clostridium sensu stricto 14*	0.28 ± 0.14	**1.55 ± 0.61**	Anal	–	–	**0.043**
*Azomonas*	0.16 ± 0.12	**1.05 ± 0.42**	Anal	3.703	**0.016**	0.053
*Dysgonomonas*	1.16 ± 0.66	**3.25 ± 0.81**	Anal	4.019	**0.023**	0.069

^*a*^ Results are shown for the host subfamilies (top), sample state of origin (middle), and secretion type (bottom). For each taxon, the average and standard deviations of abundance are shown for the two groups compared, followed by the host factor class (class) which was identified as significantly differentially abundant, and the methods’ stats, including LEfSe LDA score and *P*-value, and Metastats *P*-value. Bolded *P*-values indicate significance (*P* < 0.05), and the average abundance of the differentially abundant taxon is shown in bold text. –, Not found to be significant with LeFSe analysis.

**Fig 4:**
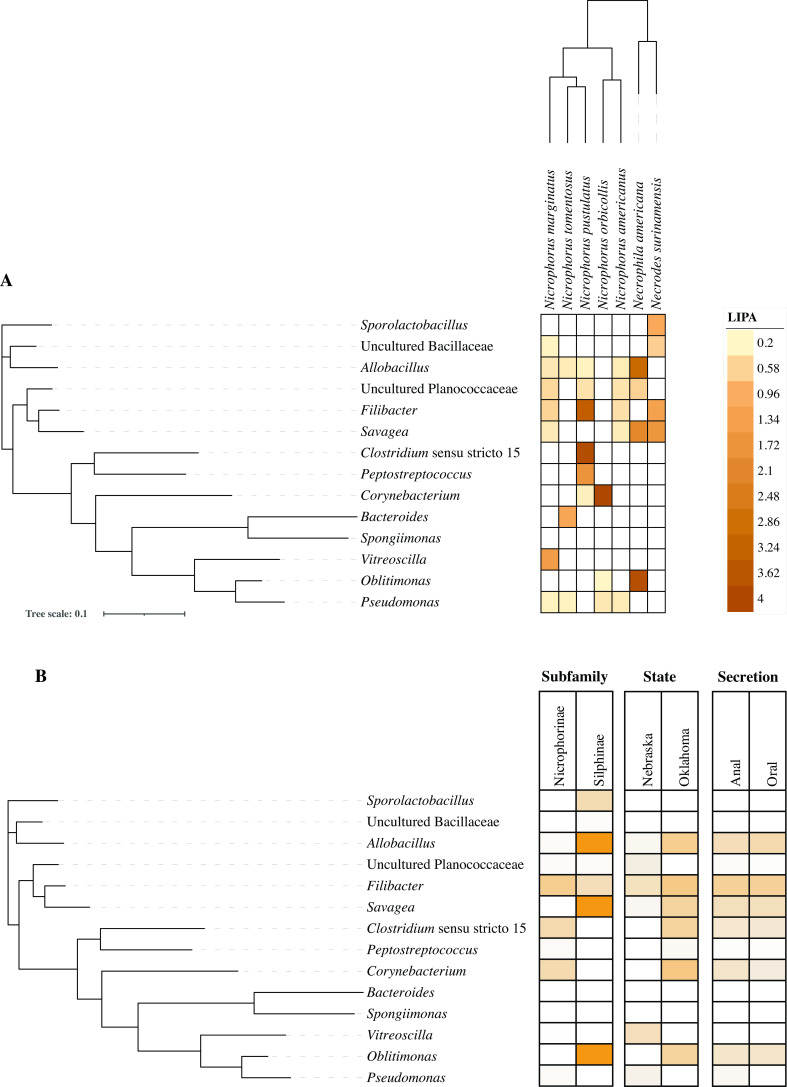
Taxa with phylogenetic signal. (**A**) The relationship between the 14 bacterial taxa with an identified phylogenetic signal is shown as a phylogenetic tree to the left. The tree was constructed from the alignment of the 16S rRNA gene sequence of the type species for each taxon. nearest alignment space termination (NAST) alignments were created in Silva, and the tree was constructed in FastTree. The heatmap is created (in iTol) using the average LIPA values as shown in the key to the right. Arbitrary increments were chosen to determine the strength of the signals, where 0.2–0.4 indicates a weak signal, 0.4–1 indicates a moderate signal, and >1 indicates a strong signal. Note that uncultured *Planococcaceae* had an Abouheif’s C_mean_ value of 0.051 and was included in this set. (**B**) The same phylogenetic tree as in (**A**) with heatmaps of LIPA values averaged for each host subfamily, each state of origin, and each secretion type.

To further assess the phylogenetic association of certain bacterial genera with the beetle species/subfamily, we calculated global phylogenetic signal statistics (Abouheif’s C_mean_, Moran’s I, and Pagel’s lambda) (Table 3). We identified 14 genera (including three core secretion microbiome genera) with significant correlations to the host phylogenetic tree (*P*-value <0.05 with at least one statistic). Based on LIPA analysis, of the above 14 genera, 13 showed significant associations with at least one beetle species (LIPA values ≥0.2), with 10 (including the three core secretion microbiome genera *Allobacillus*, *Savagea*, and *Filibacter*, in addition to *Corynebacterium*, *Peptostreptococcus, Clostridium sensu stricto 15*, *Bacteroides*, *Vitreoscilla*, *Sporolactobacillus*, and *Oblitimonas*) showing strong associations (LIPA values ≥1) with certain beetle species and three (*Savagea, Allobacillus and Oblitimonas*) showing strong associations (LIPA values ≥1) with the subfamily Silphinae (Fig. 4). Of note is the special case of *N. pustulatus*, the only species studied here with a host shift from carrion to snake eggs (13). Oral and anal secretions of *N. pustulatus* were found to be strongly associated with *Clostridium sensu stricto 15* (LIPA = 3.87), *Filibacter* (LIPA = 3.34), and *Peptostreptococcus* (LIPA = 1.72) (Fig. 4).

**TABLE 3:** Global phylogenetic signal statistics (Abouheif’s C_mean_, Moran’s I, and Pagel’s lambda) and their associated *P*-values shown for only taxa with at least one significant (*P* < 0.05) statistic^*a*^

Taxon	Abouheif’s C_mean_		Moran’s I		Pagel’s lambda	
	C_mean_	*P*-value	I	*P*-value	Lambda	*P*-value
*Sporolactobacillus*	0.042	0.095	0.141	**0.047**	0.347	0.293
Uncultured *Bacillaceae*	0.187	**0.032**	0.147	0.100	0.363	0.055
*Allobacillus*	0.475	**0.003**	0.577	**0.037**	0.627	**0.001**
*Filibacter*	0.366	**0.009**	0.655	**0.013**	0.789	**0.004**
*Savagea*	0.498	**0.001**	0.549	**0.033**	0.605	**0.001**
*Clostridium sensu stricto 15*	0.197	**0.027**	0.489	**0.002**	0.773	0.091
*Peptostreptococcus*	0.159	**0.001**	0.265	**0.001**	0.516	0.295
*Corynebacterium*	0.219	**0.020**	0.480	**0.019**	0.709	0.120
*Bacteroides*	0.127	**0.028**	0.168	**0.039**	0.387	0.436
*Spongiimonas*	0.029	**0.041**	0.048	0.297	0.189	0.455
*Vitreoscilla*	0.087	**0.015**	0.214	**0.006**	0.468	0.557
*Oblitimonas*	0.394	**0.001**	0.509	**0.027**	0.618	**0.003**
*Pseudomonas*	0.217	**0.046**	0.281	0.104	0.183	0.341

^*a*^ Significant *P*-values are shown in bold text.

## DISCUSSION

This study characterized the previously undescribed secretion microbiomes of seven species within Silphidae and identified a core microbiome comprised of 11 bacterial genera (Fig. 1). We found the host subfamily and state of origin (and to a lesser extent secretion type) to have a significant effect on the secretion microbiome community structure (Fig. 3). We identified 14 genera with significant phylogenetic association to the host subfamily, state of origin, and/or secretion type (Fig. 4; Table 3).

The ability of burying beetles to suppress the proliferation of carcass-borne microbes (22) may be a function of the relative abundances of key bacterial taxa within their guts and secretions. By harboring taxa capable of successfully colonizing carcasses during preparation, *Nicrophorus* spp. appear capable of interrupting the normal progression of decomposition and maintaining their offspring. Here, we identified a core microbiome comprised of 11 bacterial genera. However, since our experimental setup only allowed us to classify sequences down to the genus level with confidence and due to the small number of samples examined here, the actual role played by members of the silphid secretion core microbiome could only be speculated upon. The secretion core microbiome identified in this study (Fig. 1C) was generally consistent with previous research analyzing other silphid-associated microbiomes (6, 12, 22 – 26), particularly in the well-studied *Nicrophorus* spp., and thought to be likely playing a role in the preservation and digestion of carcasses (25). Many of these 11 core genera have been identified in other studies of silphid-associated microbiomes (6, 12, 22 – 26). Previous studies that isolated and characterized members of the silphid secretion core microbiome taxa have associated these members with metabolic traits that could explain their role in providing a benefit to the host. For example, the fatty acid degrading *Dysgonomonas* spp., the ammonifying *Clostridium* spp., and the urease-producing *Ignatzschineria* spp. (6) may provide host benefits by making nutrients available to the host (6, 9, 12). Similarly, the genera *Vagococcus, Clostridium,* and *Tissierella* (all are anaerobic Firmicutes that ferment creatinine, a metabolite abundant in animal tissues, but cannot be utilized by insects as a sole carbon and energy source) could also be beneficial to the host (6). On the other hand, members of other core microbiome taxa have not been reported in previous studies of silphid-associated microbiomes. However, some of the characteristics of members of these taxa could explain their presence in silphid secretions as likely carcass preservatives. For example, members of the genus *Carnobacterium* have been studied as protective cultures for food (27). Finally, members of other core microbiome taxa are poorly characterized [e.g., *Filibacter* (28, 29), *Allobacillus* (30), *Savagea* (31, 32), candidate genus *Soleaferrea* (33), and *Sphingobacterium* (34)], making speculations about their possible role in silphid secretions difficult without further studies. It is worth noting that while we focused here on the bacterial community of secretions, a fungal component of the secretion microbiome cannot be ignored, as previous research on silphid gut microbiome identified both fungal and bacterial communities (6, 12). Identifying the silphid secretion mycobiome is a topic for future research.

Host phylogeny is a strong driver of the microbiome in mammals (35, 36), insects (37), birds (38), and other Metazoa (39, 40). Here, we showed that the beetles’ secretion bacterial community was shaped by the beetle subfamily (Fig. 3; Fig. S2), and that many of the core microbiome genera were significantly more abundant in one host subfamily (Table 2). This is in contrast to previous studies that compared the gut microbiomes of the two silphid subfamilies (6). Our global and local phylogenetic association analyses identified 13 genera (including three of the core genera) as having a significant association with the host phylogeny (Table 3; Fig. 4). The genera *Corynebacterium*, *Peptostreptococcus*, *Pseudomonas,* and *Vitreoscilla* showed strong associations with the subfamily Nicrophorinae*,* all of which are known to be host-associated (41 – 44). On the other hand, in addition to the three core secretion microbiome genera *Allobacillus*, *Savagea*, and *Filibacter* that all showed a high phylogenetic signal to the Silphinae subfamily, the two genera, *Sporolactobacillus* and *Oblitimonas,* showed significant phylogenetic association with *Necrodes surinamensis* and *Necrophila americana*, respectively. These two genera were not previously reported as members of the Silphinae gut microbiome. *Sporolactobacillus* is rarely isolated from host-associated sources (45), while *Oblitimonas* isolates commonly associate with human clinical samples (46), but have not been reported in meat or associated with other hosts. The unique case of *N. pustulatus* is noteworthy. This is the only species studied here with a host shift from carrion to snake eggs (13). LIPA analysis showed uniquely high values of association of *N. pustulatus* oral and anal secretions with *Filibacter*, *Peptostreptococcus*, and *Clostridium sensu stricto 15*. Previous research showed that *N. pustulatus* secretions did not cause significant reductions in bacterial growth (antimicrobial activity) when compared to secretions from other carrion beetles of the subfamily Nicrophorinae (10). Whether the unique secretion microbiome of *N. pustulatus* explains these results is a subject for future studies.

Geographical location was shown to have an effect on gut microbiome (47 – 50). Kaltenpoth and Steiger (6) compared the gut microbiomes of the two silphid subfamilies, and highlighted geographical patterns in microbial communities of *Nicrophorus* spp. We showed here that geographical regions significantly influenced bacterial communities within the secretions of silphids (Fig. 3; Fig. S2). Our LEfSe and Metastats analyses identified the core genera *Filibacter* and *Savagea* to be significantly more abundant within the secretion bacterial communities of beetles from Oklahoma, and the core genus *Sphingobacterium* to be significantly more abundant within the secretion bacterial communities of beetles from Nebraska (Table 2). In addition to core genera, the genera *Proteus, Enterococcus, Spongiimonas,* and *Acinetobacter* were also identified as differentially abundant in the two states (Table 2). Members of *Proteus, Enterococcus,* and *Acinetobacter* have previously been identified in a number of other silphid-associated microbiome studies (6, 9, 12, 22 – 26). Phylogenetic signal analysis using LIPA identified *Savagea*, *Corynebacterium*, *Clostridium sensu stricto 15*, *Allobacillus*, and *Oblitimonas* to have a higher phylogenetic signal in Oklahoma samples, and *Vitreoscilla* to have a higher phylogenetic signal in Nebraska samples (Fig. 4). However, we acknowledge that this effect could be biased as all the Silphinae samples were collected from Oklahoma, with no Nebraska Silphinae tested.

Previous studies identified antibiotic-producing bacteria in silphid gut (51), and others showed that these antibiotic-producing bacteria can be transmitted to carcasses through anal secretions (9) to help eliminate nonessential or even pathogenic bacteria from carcasses. Hoback et al. (10) identified differences in antimicrobial activities between oral and anal secretions of carrion beetles. Notably, this study is the first to characterize both the oral and anal secretion microbiomes of silphid species. We showed that the secretion source has a significant, albeit smaller, effect on community structure [when using Bray-Curtis as the measure for beta diversity (Table 1)]. More importantly, our LEfSe and Metastats analyses identified the core genera *Ignatzschineria, Candidatus Soleaferrea, Tissierella, Clostridium sensu stricto 14,* and *Dysgonomonas* to be more significantly abundant in anal secretions compared to oral secretions (Table 2). It is possible that the differences in antimicrobial activities previously observed between anal and oral secretions (10) could potentially be explained by the difference in relative abundance of these bacterial genera. Future culture-dependent studies from silphid secretions may identify novel antimicrobials and nontoxic compounds that can act as meat preservatives.

Diet is among other factors that could potentially shape the secretion microbiome of silphids. Studies that compared gut microbiomes of insects [e.g. reference (37)] showed a strong clustering effect driven by diet. Carrion beetles’ reproductive success and larval care were found to vary on fresh and old carcasses (52). Such effect on the beetles’ secretion microbiome is not well understood and should be the topic for future studies.

Description of the bacterial communities present in silphid secretions furthers our understanding of how these beetles interact with microbes for carcass nutrient processing. Testing secretions from two subfamilies that differ in reproductive strategies also allows further insights into selection and adaptation by Nicrophorinae.

## Data Availability

Illumina reads are deposited in GenBank through BiopProject accession number PRJNA892615, BiosSample accession numbers SAMN31390780-–SAMN31390799, and Short Read Archive (SRA) accession number SRR21986868- –SRR21986887.

## SUPPLEMENTAL MATERIAL

Supplemental material spectrum.01694-23-s0001.docx Table S1 and Figures S1, S2, and S3.

ASM does not own the copyrights to Supplemental Material that may be linked to, or accessed through, an article. The authors have granted ASM a non-exclusive, world-wide license to publish the Supplemental Material files. Please contact the corresponding author directly for reuse.
