# Supplementary material for: Culture-independent analyses of carrion beetle (Coleoptera: Silphidae) secretion bacterial communities
Source: Microbiol Spectr. 2023 Oct 24;11(6):e01694-23. doi: 10.1128/spectrum.01694-23 (PMC10714842; doi:10.1128/spectrum.01694-23)
Supplement: Supplemental material — Table S1 and Figures S1, S2, and S3. [file spectrum.01694-23-s0001.docx]

Supplementary data for:

**Culture independent analyses of carrion beetle (Coleoptera: Silphidae) secretion bacterial communities**

Carrie J. Pratt^1^, Casey H. Meili^1^, Noha H. Youssef^1*^, W. Wyatt Hoback^2^

^1^ Department of Microbiology and Molecular Genetics, Oklahoma State University, Stillwater, Oklahoma, United States of America

^2^ Department of Entomology and Plant Pathology, Oklahoma State University, Stillwater, Oklahoma, United States of America

* Corresponding author

E-mail: noha@okstate.edu

Table S1. Details of samples collected for this study.

| Beetle species | Number of beetles | Individuals pooled? | Beetle sex | Collection state |
| --- | --- | --- | --- | --- |
| *Necrodes surinamensis* | 5 | yes | mixed | OK |
| *Necrophila americana* | 5 | yes | mixed | OK |
| *Nicrophorus americanus* | 4 | no | M (1) & F (1) | NE |
|  |  |  | M (1) & F (1) | OK |
| *N. marginatus* | 5 | yes | mixed | NE |
| *N. orbicollis* | 4 | yes | mixed | OK |
| *N. pustulatus* | 4 | yes | mixed | OK |
| *N. tomentosus* | 4 | yes | mixed | OK |

**Figure S1**. Timetree (timetree.org) tree highlighting the relationship between the twenty samples studied here. The tree was downloaded from timetree.org and modified to include all the samples studied here with very short branch length between samples from the same beetle species. Species are color labeled as shown in the legend, and the silphid subfamily, secretion type, sex, pooling, state of origin, and number of individuals per pool are shown to the right of the tree.


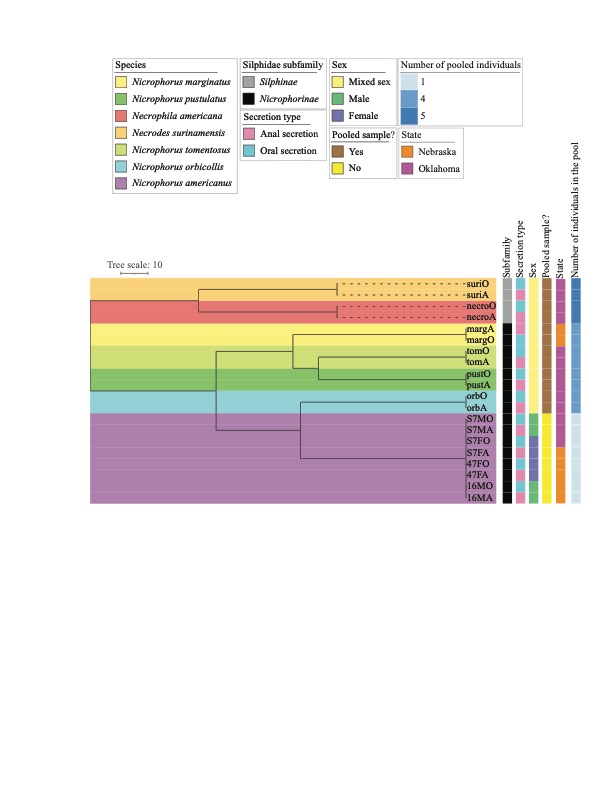


**Figure S2.** Rarefaction curves showing the increase in the number of genera observed as the number of sequences increase per sample. Samples are named similar to Figure S1.

**Figure S3.** Bacterial community structure in the samples studied. Ordination biplots (both PCoA and NMDS) constructed with both dissimilarity matrix-based (Bray-Curtis (A & B)) and phylogenetic similarity-based (weighted Unifrac (C & D)) indices describe the similarity between communities. The percentage variance explained by the first two PCoA axes are shown on the corresponding axis. Samples are color coded by the state of origin, while the shape depicts the host subfamily as shown on top. The 64 most abundant bacterial taxa are shown as squares, where the eleven genera constituting the core microbiome, and the fourteen taxa with phylogenetic signal are shown as filled squares. Core taxa that were also shown to have phylogenetic signal (n=3) are color-coded in purple. Other core taxa are color-coded in red, while other taxa with phylogenetic signal are color-coded in cyan.
